# High-density QTL mapping of leaf-related traits and chlorophyll content in three soybean RIL populations

**DOI:** 10.1186/s12870-020-02684-x

**Published:** 2020-10-13

**Authors:** Kaiye Yu, Jinshe Wang, Chongyuan Sun, Xiaoqian Liu, Huanqing Xu, Yuming Yang, Lidong Dong, Dan Zhang

**Affiliations:** 1 National Key Laboratory of Wheat and Maize Crop Science, Collaborative Innovation Center of Henan Grain Crops, Agronomy College, Henan Agricultural University, Zhengzhou, 450002 Henan China; 2 Zhengzhou National Subcenter for Soybean Improvement, Henan Academy of Agricultural Sciences, Zhengzhou, 450002 Henan China; 3 School of Life Sciences, Guangzhou University, Guangzhou, 510006 Guangdong China

**Keywords:** Soybean, Leaves related traits, Chlorophyll content, Quantitative trait loci, Genetic relationship

## Abstract

**Background:**

Leaf size and shape, which affect light capture, and chlorophyll content are important factors affecting photosynthetic efficiency. Genetic variation of these components significantly affects yield potential and seed quality. Identification of the genetic basis for these traits and the relationship between them is of great practical significance for achieving ideal plant architecture and high photosynthetic efficiency for improved yield.

**Results:**

Here, we undertook a large-scale linkage mapping study using three mapping populations to determine the genetic interplay between soybean leaf-related traits and chlorophyll content across two environments. Correlation analysis revealed a significant negative correlation between leaf size and shape, while both traits were positively correlated with chlorophyll content. This phenotypic relationship was verified across the three mapping populations as determined by principal component analysis, suggesting that these traits are under the control of complex and interrelated genetic components. The QTLs for leaf-related traits and chlorophyll are partly shared, which further supports the close genetic relationship between the two traits. The largest-effect major loci, *q20*, was stably identified across all population and environments and harbored the narrow leaflet gene *Gm-JAG1* (*Ln*/*ln*), which is a key regulator of leaflet shape in soybean.

**Conclusion:**

Our results uncover several major QTLs (*q4–1*, *q4–2*, *q11*, *q13*, *q18* and *q20*) and its candidate genes specific or common to leaf-related traits and chlorophyll, and also show a complex epistatic interaction between the two traits. The SNP markers closely linked to these valuable QTLs could be used for molecular design breeding with improved plant architecture, photosynthetic capacity and even yield.

## Background

Soybean is an important crop that provides oil and protein to the global population. In recent years, the global demand for soybean has increased rapidly. Therefore, increasing yield is one of the most important goals of soybean breeding programs. The yield of crops largely depends on leaf photosynthetic capacity. Crop yield and quality are also influenced by leaf-related traits, such as leaf shape, which not only affect light penetration, light absorption, CO_2_ fixation and photosynthetic efficiency, but also the canopy structure of the population, thus determining the light distribution, light energy utilization efficiency and ventilation permeability [1]. In soybean, leaf width (LW), leaf length (LL), and leaf area (LA) are important components of plant architecture; optimizing these leaf shape characteristics can improve the geometry and spatial arrangement of leaves, achieve the ideal plant canopy shape, reduce individual shading response, and improve the photosynthetic efficiency of leaves and yield [2]. Chlorophyll content (CC) is also an important factor affecting photosynthetic efficiency, biomass and yield in crops [3–6] and has been used to estimate leaf photosynthetic efficiency and yield potential in rice [7]. A high CC is a desired characteristic because it indicates that the degree of photoinhibition in photosynthesis is low [8]. Therefore, revealing the genetic relationships and epistasic interactions between leaf-related trait and CC QTLs and their interactions with the environment is of great practical significance for breeding soybean with high photosynthetic efficiency and high yield.

At present, although many QTLs related to leaf-related traits and CC have been identified in soybean (https://www.soybase.org/), the genetic relationship between the two traits, including epistasic and environmental interaction effects, has not been reported. Moreover, studies identifying QTLs for soybean leaf-related traits and CC were performed separately, and were limited by the narrow genetic background of the isolated populations and the use low-resolution molecular markers. A previous study reported that a lot of QTLs related to leaf traits co-localize with QTLs for CC in wheat [9–11]. Co-localization of multiple QTLs is associated with the genetic correlation between phenotypes, and also indicates the possibility of multiple gene linkages or multiple effects. Therefore, identification of QTLs/candidate genes controlling leaf-related traits and CC and the genetic relationships between them not only can provide guidance for breeding soybean for improved plant architecture but also can be important for improving photosynthetic efficiency and even yield.

To gain deeper insights into the genetic structure of variation in leaf-related traits and CC, we exploited three recombinant inbred lines (RILs) populations, which extensive capture of phenotypic variation in soybean germplasm pool to map QTLs for LA, LL, LW, L/W (the ratio of leaf length and width), and CC across multiple environments using high-density genetic maps, and also analyzed the 100-seed weight per plant (100-SW) for reference and comparison. The aims of this study were to i) analysis the phenotypic relationship between leaf-related traits and CC using three RIL populations grown across multiple environments, ii) identify the genetic structure of the relationship between leaf related-traits and CC by using QTL mapping, iii) identify major QTLs that are stable in multiple environments, iv) identify molecular markers associated to valuable QTLs, which may be beneficial in improving both plant architecture and photosynthetic capacity, and v) predict potential candidate genes responsible for valuable QTLs. The results showed that several loci should be useful tools for the genetic improvement of photosynthetic efficiency and yield related traits in soybean.

## Results

### Leaf-related traits and chlorophyll content exhibited significant phenotypic variation in three soybean RIL populations

**Fig. 1:**
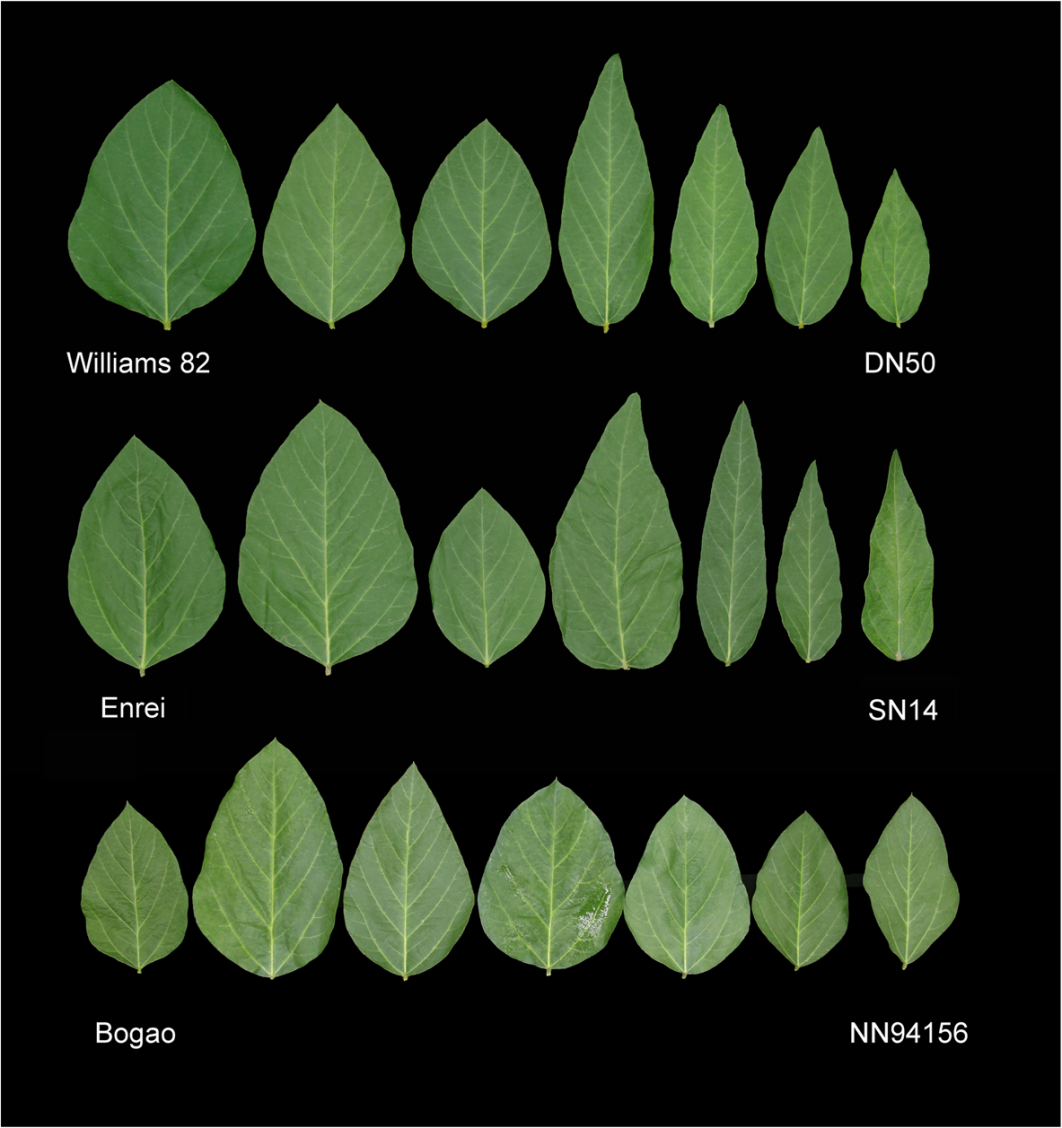
Phenotypic variation in leaf size and shape among parents and progeny in three RIL mapping populations. The female parents, Williams 82, Enrei and Bogao had a leaf length/width ratio of ~ 1.5, while the male parents, Dongnong50 (DN50), Suinong 14 (SN14) and Nannong 94–156 (NN94156) had a ratio of ~ 3.0. The segregating progeny exhibit transgressive segregation in leaf length/width ratio, as this ratio ranged from about 0.95 to 4.32

**Fig. 2:**
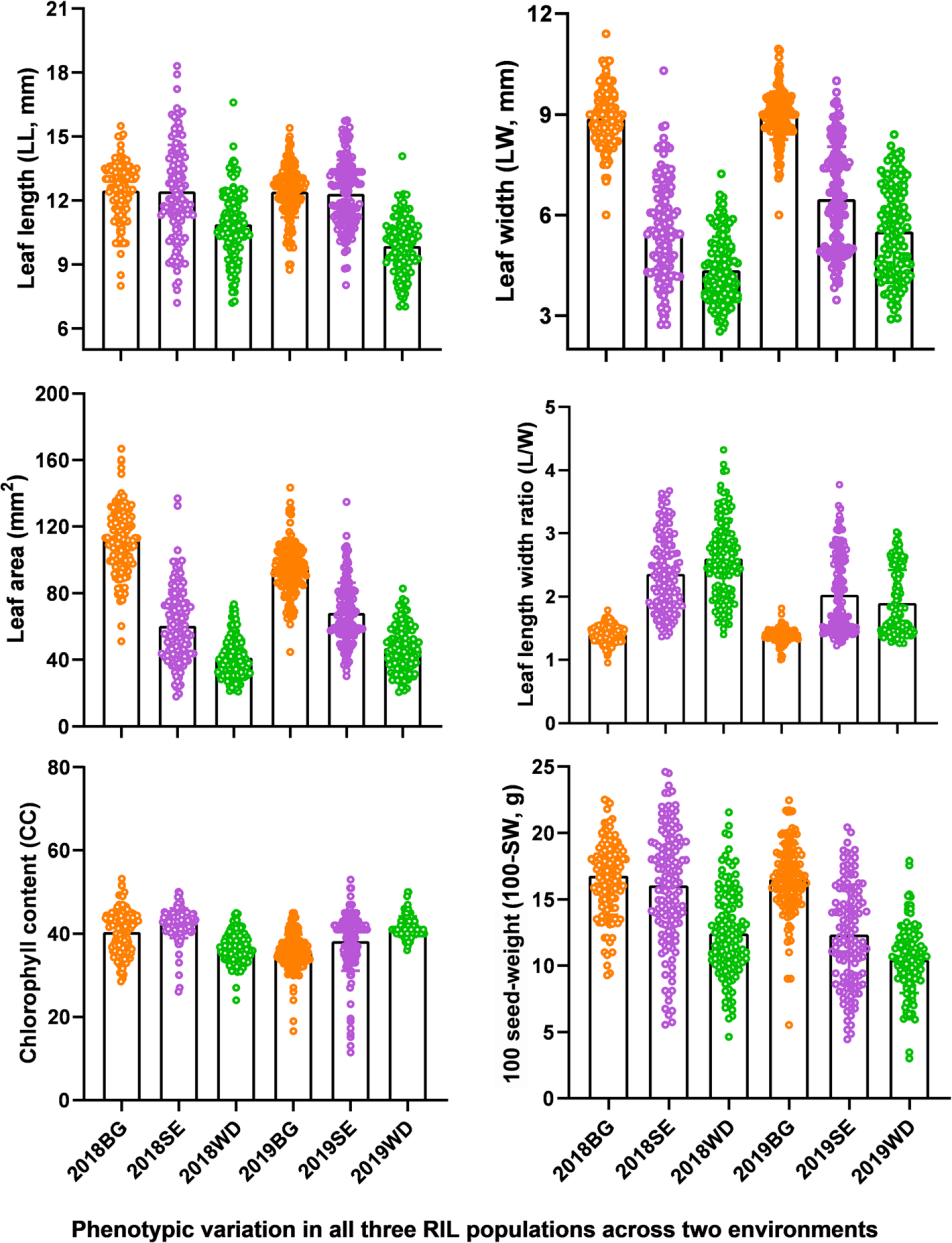
Phenotypic analysis of leaf-related traits, chlorophyll content and seed weight in all three RIL populations across two environments. Bar plots represent the mean value of phenotype data. The colored dots represent individual data points. BG, SE, and WD denote the mapping populations and 2018 and 2019 denote the environments (years) in which the populations were grown (Drawn by GaphPad Prism 8.0.2)

A total of six parameters, LL, LW, LA, L/W, CC, and 100-SW, were measured to determine the variation of leaf size, shape, photosynthetic capacity, and yield related traits potential in a collection of three RIL mapping populations grown across two environments (Fig. 1, Table 1). Except for Nannong94–156 and Bogao, which had no obvious difference in LW or LA, the parental lines exhibited significant differences for all these traits (Table S1). In addition, there was extensive transgressive segregation for all six traits in all three RIL populations, with some descendant lines showing superior phenotypic values to their parents (Figs. 1 and 2, Figs. S1 and Table S1). The phenotypic values of descendant lines ranged from 7.86–13.43 cm for LL, 3.83–9.13 cm for LW, and 24.32–92.49 cm^2^ for LA (Table S1). The mean CC values for the RILs ranged from 7.19–53.23, and the mean 100-SW values ranged from 3.02–28.44 mg. Among the diverse soybean lines, the highest L/W ratio was 3.04; however, one soybean RIL had a ratio of only 1.36 (Table S1). Overall, the soybean lines clearly exhibited considerable natural variation in traits related to leaf size, shape and chlorophyll and displayed very high genetic diversity. The observation of transgression shows the polygenic inheritance of leaf related-traits and CC with both parents contributing to increased and decreased trait alleles. Among RIL lines, significant differences were found for all six traits in each individual population (*P* < 0.01). Moreover, we observed significant genotypic and environmental effects for all populations and traits within and between years. The broad sense heritability of all traits was moderate to high, ranging between 0.59 and 0.89, and L/W showed the highest heritability (*h*^*2*^ = 0.81–0.89) across all populations (Table S1).

**Table 1:** Information on the three RIL mapping populations

Populations	Parents	Abbreviation	Number of Lines	Environments^**a**^
RIL populations	Williams82 × Dongnong50	W × D	127 RIL	2018, 2019
	Suinong14 × Enrei	S × E	146 RIL	2018, 2019
	Nannong94–156 × Bogao	N × B	156 RIL	2018, 2019

^a^The years of which each experiment was carried out

### Phenotypic structure of leaf-related and chlorophyll traits

Pairwise analyses of the six traits using simple linear correlation coefficients (Pearson’s correlation) indicated that the most leaf-related traits, CC, and 100-SW were significantly correlated (*P* < 0.05 or 0.01) with each other in all three RIL populations and in both years (Table S2). These results suggest that leaf-related traits and CC could be important factors affecting soybean yield related traits. LL, LW, and LA were all positively correlated with 100-SW, with the strongest correlation (*r* = 0.23–0.34) identified between LL and 100-SW, suggesting that soybean yield related traits was most affected by LL. In addition, the leaf-related traits were also inter-correlated to various degrees. For example, LA was highly positively correlated (r ≥ 0.86, *P* < 0.01) with LW and moderately correlated with LL (r ≥ 0.37, *P* < 0.01) in all three populations and in both years, suggesting that LA is mainly determined by LW. Interestingly, LW was significantly negatively correlated with L/W, which had a very weak correlation or no significant with either CC and 100-SW (Table S2). These results suggested that the L/W ratio, which largely describe leaf shape, are independent of CC, 100-SW, photosynthesis and yield. In summary, the results of the correlation analysis showed that LL has a positive effect on CC and 100-SW.

**Fig. 3:**
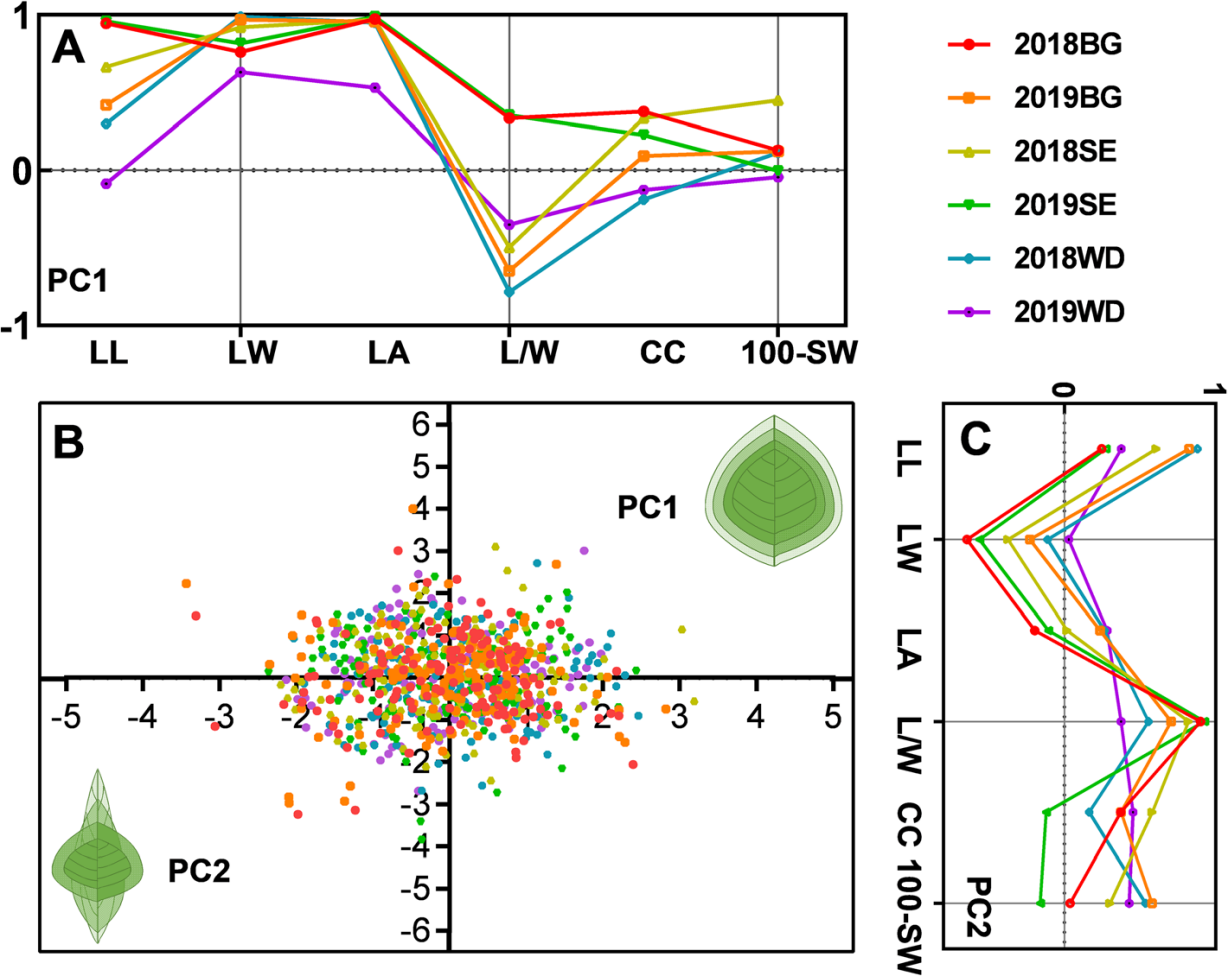
A morphometric model for variation in leaf-related traits, chlorophyll content and 100-seed weight in three soybean RIL populations. (A) and (B) Variation in leaf size is captured by PC1 with both leaf length and width having large effects, whereas PC2 describes variation in leaf shape largely through changes in leaf length and the ratio of leaf length to width. Component loading (i.e., correlations between the variables and factors) for PC1 (A) and PC2 (C) for each population are color-coded. (B) Score distribution for PC1 and PC2. Schematic representation of variation in leaf size and shape captured by PC1 (x-axis) and PC2 (y-axis), respectively (Drawn by GaphPad Prism 8.0.2)

To dissect the major sources of variation in the phenotypes in each RIL population and in the entire population (Table S3), we performed a principal component analysis (PCA), taking into account the complex interrelationships among various phenotypic traits. In the present study, only variables with loading values > 0.4 were considered important. Two significant principal components (PCs), including PC1 and PC2 were extracted for each RIL population, and these PCs capture 71.3 to 75.8% of the phenotypic variation across three populations (Table S3). PC1 and PC2 showed similar structure in all three mapping populations; PC1 (44.7 to 46.5%) and PC2 (26.4 to 29.3%) primarily accounted for variation in leaf size (LW, LA, and LL) and leaf shape (LL, L/W), respectively (Fig. 3A and C). Therefore, PC1 mainly explained the differences of leaf size, and the increase of proportion along the length and width axes was positively correlated with the increase of LA and 100-SW (Fig. 3B). In contrast, PC2 primarily captures the differences of leaf shape (Fig. 3C), among which L/W ratio and LL being the main explanatory factors. Interestingly, PC1 and PC2 also capture a portion of the variation in CC and 100-SW (Fig. 3A and C). Overall, the close phenotypic relationships of leaf-related traits and CC with yield related traits was captured by PCA in the three RIL populations, indicating that these traits are under the control of complex and interrelated genetic components.

### The variation of genetic structure is consistent with that of phenotypic variation

**Fig. 4:**
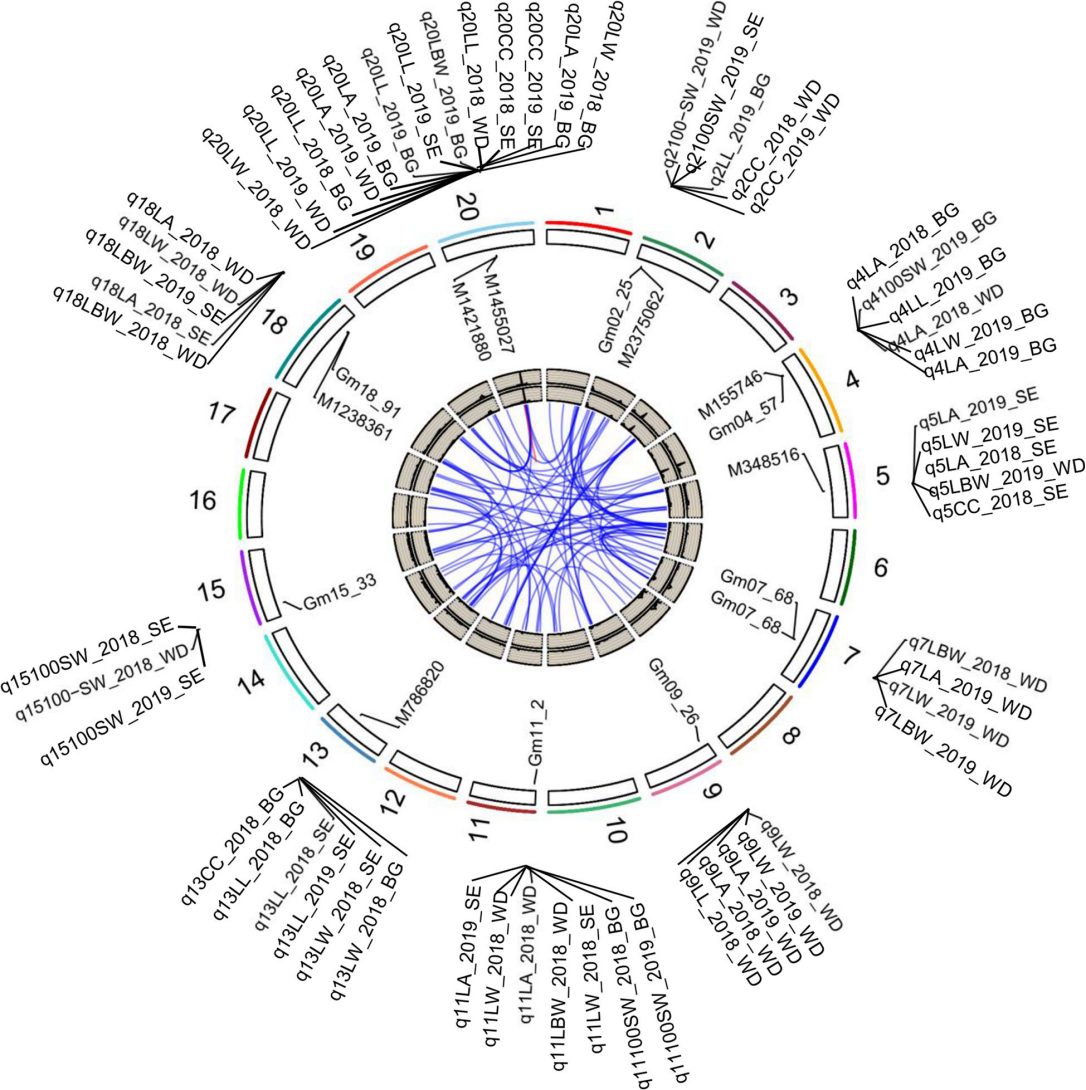
QTLs for soybean leaf-related traits, chlorophyll content and 100-seed weight identified on soybean chromosomes by linkage mapping in three RIL populations. The lines linking loci denote epistatic associations between QTLs. Blue lines denote links between two QTLs on different chromosomes, while red lines denote links between two QTLs on the same chromosome. The outside/inside wheat-colored circle indicates the LOD/percent variance explained values for the investigated traits across environments. The outermost circle indicates the 20 soybean chromosomes, QTLs for investigated traits, and the positions of linked markers for these QTLs on the chromosomes (Drawn by R 3.6.2)

Given the phenotypic model for the leaf size, shape, CC and 100-SW parameters (Table S2, Fig. 3), we hypothesized that these traits may be inherited dependently under the control of different genetic components. In order to test this hypothesis, we conducted QTL analysis using the data for leaf-related traits, CC and 100-SW in the three RIL populations across two different environments. A high-density genetic map for each population was used; these maps were constructed using 6159 SNPs for N × B [12], 5660 bin markers for S × E and 2015 bin markers for W × D. Consistent with the observation of extensive transgressive segregation (Figs. 1 and 2, Figs. S1), the leaf traits were quantitatively inherited in the RIL populations. A total of 96 QTLs (40 QTLs in W × D, 32 QTLs in N × B, and 26 QTL in S × E) were identified across traits, environments (years) and mapping populations (Fig. 4, Table 2 and Table S4). The LOD scores for each of these QTLs ranged between 2.0 and 22.6 and explained 5.6 to 42.4% of the phenotypic variation. In general, nearly one third of these QTLs were pleiotropic, affecting leaf-related traits and CC, consistent with the close correlation among these tested traits (Table 2). For example, QTL *q20* simultaneously controls leaf size, shape and CC across populations and environments (Table 2, Fig. 4). Meanwhile, we also observed that several QTLs were population-specific or environment-specific, suggesting that the underlying variation may either exist only in a certain population or be sensitive to the environment. For example, in the W × D RIL population, 40 QTLs were identified on 15 chromosomes for all selected traits in across experiments (Table S4). Among the 40 QTLs, 22 were detected only in 2018 while 18 were only detected in 2019 (Fig. 4, Table 2, and Table S4). The percent phenotypic variation explained by these QTLs ranged from 2.74% (*q5L/W4_2019_WD*) to 42.44% (*q20L/W4_2019_WD*) with the LOD values ranging from 2.11 to 22.59.

**Table 2:** The characteristics of 25 consensus loci associated with leaf-related traits, chlorophyll content and 100-seed weight across years and mapping populations

Name^**a**^	Traits-years-populations^**b**^	Chr.^**c**^	Marker interval^**d**^	Position^**e**^	LOD^**f**^	PVE(%)^**g**^
*q1*	LW4_2019_SE, LW4_2019_SE	1	Marker78716-Marker78952	984,367–984,667	2.86	12.28
*q2–1*	LL_2019_BG, 100-SW_2019_WD	2	Marker2375062-M2398952	5,036,099–5,089,021	2.76	8.77
*q2–2*	100SW_2019_SE, CC_2018_WD, CC_2019_WD	2	Gm02_72-Gm02_73	15,948,773–20,504,503	4.02	9.16
*q3–1*	LA_2019_BG, CC_2018_BG, CC_2019_BG	3	Marker946135-Marker945189	18,840,357–18,840,627	5.57	11.08
*q3–2*	CC_2018_WD, CC_2019_WD, LW_2019_WD 100SW_2019_BG	3	Marker974279-Marker963484	38,833,014–38,833,279	3.66	6.24
***q4–1***	**100SW_2019_BG, LA_2018_WD, CC_2019_BG, 100SW_2018_BG, L/W_2018_WD**	4	Marker155746-Marker7176	8,832,243–8,832,495	3.69	6.60
***q4–2***	**LW_2019_BG, LA_2018_BG, LL_2019_BG, LA_2019_BG, LA_2018_WD, CC_2018_BG**	4	Marker56301-Marker85908	40,171,476–40,171,747	3.28	8.30
*q5–1*	LA_2019_SE, LW_2019_SE, LA_2018_SE	5	Marker348516-Marker349213	31,386,634–31,386,733	3.26	8.01
*q5–2*	L/W_2019_WD, CC_2018_SE	5	Gm05_82-Gm05_83	41,012,328–41,053,017	2.64	2.74
*q6*	L/W_2019_WD, CC_2018_BG	6	Gm06_25-Gm06_26	8,267,811–8,524,123	4.11	4.39
*q7*	L/W_2018_WD, LW_2019_WD, LA_2019_WD, L/W_2019_WD	7	Gm07_68-Gm07_69	26,688,332–26,773,863	3.66	8.18
*q8*	CC_2018_BG, CC_2019_BG, LL_2019_BG	8	Marker2655981-Mrker2673143	1,796,491–1,796,812	3.05	5.85
*q9–1*	LW_2018_WD, LW_2019_WD, LA_2019_WD	9	Gm09_27-Gm09_28	4,248,062–4,381,966	3.24	7.11
*q9–2*	LA_2018_WD, LL_2018_WD	9	Gm09_92-Gm09_93	38,324,753–38,453,818	6.44	16.25
*q10*	LL_2018_WD	10	Gm10_28-Gm10_29	5,215,402–5,261,825	3.06	7.26
***q11–1***	**LA_2018_WD, LW_2018_WD, L/W_2018_WD, LW_2018_SE, LA_2019_SE**	11	Marker649826-Marker649720	5,906,321–5,906,420	3.15	6.00
*q11–2*	100SW_2018_BG, 100SW_2019_BG	11	Marker789315-Marker822756	24,450,424–24,450,687	8.52	23.80
*q12–1*	CC_2018_BG, CC_2019_BG, L/W_2018_SE	12	Marker179649-M195752	3,339,466–3,339,756	5.04	10.00
*q12–2*	CC_2019_WD, 100-SW_2019_WD	12	Gm12_53-Gm12_54	19,741,984–19,784,797	3.12	10.88
***q13***	**LL_2018_SE, LW_2018_SE, LW_2018_BG, LL_2019_SE, CC_2018_BG, LL_2018_BG**	13	Marker1729776-Marker1800330	35,339,389–35,339,673	3.23	8.47
*q14*	100-SW_2018_WD, LL_2018_BG	14	Marker562393-Marker489485	3,347,418–3,347,071	3.56	9.03
*q15*	100-SW_2018_WD, 100SW_2018_SE, 100SW_2019_SE	15	Marker888970-Marker891668	11,184,661–11,184,760	3.12	9.92
*q16*	CC_2018_WD, CC_2019_WD	16	Gm16_62-Gm16_63	32,211,900–32,464,530	3.88	12.13
***q18***	**LA_2018_SE, LW_2018_WD, LA_2018_WD, L/W_2018_WD, L/W_2019_SE**	18	Gm18_91-Gm18_92	55,902,104–56,060,463	10.22	25.16
***q20***	**LL_2019_BG, CC_2018_SE, CC_2019_SE, LL_2018_WD, LW_2019_WD, LA_2019_WD, L/W_2019_WD, LA_2019_SE, LW_2018_SE, L/W_2018_SE, LL_2019_SE, LW_2019_SE, L/W_2019_SE, LA_2018_BG, LW_2019_BG, LA_2019_BG, LW_2018_BG, LL_2018_BG, LW_2018_WD, L/W_2018_WD, LL_2019_WD**	20	Gm20_56-Gm20_57	35,359,456–35,474,870	32.59	42.44

^a^The name of the QTL is defined by the chromosome number

^b^The name of the QTLs is a composite of the target trait [leaf length (LL), leaf width (LW), leaf area (LA), leaf length to width ratio (L/W), chlorophyll content (CC), and 100 seed weight (100 SW)], followed by the environment (year), and mapping population

^c^Chr indicates chromosome. Major QTLs are shown in bold

^d^Interval indicates the confidence interval between two markers

^e^Position indicates the physical position of the interval in the soybean genome

^f^LOD is the average logarithm of odds score

^g^PVE is the average phenotypic variance explained by the QTL

### Determination of major and co-localized loci associated with leaf-related traits and chlorophyll content

Previous studies reported that lead SNPs less than or around 5 Mb apart were thought to be caused by a single locus that affect the trait [13]. According to this criterion, 96 QTLs were classified into 25 loci (Table 2 and Table S4), and almost all were found to be pleiotropic, which was consistent with a significant correlation of phenotypic traits. Furthermore, we found that where the broad-sense heritability of a trait was very high (e.g., L/W), some major QTLs (such as *q18* and *q20*) are common in both years and in all given populations (Fig. 4, Table 2 and Table S4). Further analysis of the 25 loci showed that six could be identified (more than five times) repeatedly across traits, years or populations. Then the six loci, *q4–1, q4–2, q11–1, q13, q18*, and *q20*, were considered as major or stable QTLs (Fig. 4, Table 2 and Table S4).

The six major QTLs, which were distributed on chromosomes 4, 11, 13, 18, and 20 (Table 2), had average LOD score of 5.96 and explained appromixately 13.16% of phenotypic variance (Table 2, Table S5). In addition, comparative analyses showed that three QTLs (*q11*, *q18*, and *q20*) were co-localized with previously identified leaf-related QTLs identified in natural populations by genome-wide association studies (GWAS) [14, 15]. It is worth noting that these three loci were identified across traits, years and populations through linkage mapping in the present study, suggesting that these loci might play important roles in leaf-related traits, CC and even yield in soybean. Among the three loci, *q20* was the largest QTL cluster harboring 21 QTLs associated with all the leaf-related traits (LL, LW, LA and L/W) and CC across years or populations. The LOD score of this locus was 9.27 on average (Fig. 4, Table 2, and Table S4), which could explain 19.74% of the phenotypic variation on average. Moreover, *q20* was co-localized with the *Ln* locus (*Gm-JAG1*), an important regulator of leaflet shape [16].

Interestingly, the other two major QTLs, *q11*and *q18*, were both associated with LW, LA and L/W across years and populations, consistent with the results of correlation analysis mentioned earlier where LA and L/W were highly correlated with LW. Further analysis of these two QTLs revealed that L/W-related loci presented positive additive effects, while LW- and LA-related loci presented negative additive effects across the 2 years and populations, which is consistent with the positive and negative correlation between phenotypic traits. Therefore, QTLs such as *q11*, *q18,* and *q20*, which had high LOD values and explained a high percentage of the phenotypic variation, may be the key QTL hotspots contributing to leaf-related traits and CC.

Another three loci (*q4–1, q4–2,* and *q13*) were not reported in previous studies, and represent novel loci controling soybean leaf-related traits and CC. For example, the novel major QTL, *q13*, was associated with LL, LW, and CC across years and populations, suggesting that LL, LW and CC may be controlled by common genes in soybean. The LOD of this locus was 5.52 on average, and *q13* could explain 5.61–25.17% of the phenotypic variance (Table S4). Interestingly, we found the two novel major loci, *q4–1* and *q4–2*, were both linked to leaf-related traits, CC and yield related traits, suggesting that these two loci may have important effects on soybean photosynthesis and even yield. More important, all the valuable QTL alleles of *q4–1* and *q4–2* were come from the male parent (DN50, Suinong 14, and Bogao) with a larger LL or L/W ratio. These results show that *q4–1* and *q4–2* could be effectively applied to soybean breeding and improve the photosynthetic capacity and even yield. Moreover, these results indicated that QTL mapping of multiple populations in multiple environments using high-density genetic maps is an effective strategy to identify major and stable QTLs at whole genome-wide.

### Epistatic QTLs for leaf-related traits, chlorophyll content and 100-seed weight

Given that leaf-related traits, CC, and 100-SW are complex traits, epistatic effects between different QTLs may exist. Additionally, among the 25 identified loci, 10 loci were detected only in one mapping population, and five QTLs were detected only in one environment, suggesting that these QTLs may interact with the environment. Therefore, besides the additive effect of QTLs, we also identified epistatic effects of QTLs for the six traits in this study. As a result, epistatic interactions between a total of 74 pairs of QTLs on all 20 chromosomes (LOD > 4.0) were identified across different populations. These QTLs explained 2.22–19.25% (Fig. 4, Table S5) of the phenotypic variation. There were 21 pairs in W × D, 29 pairs in N × B, and 24 pairs in S × E were identified across traits, years and mapping populations. There were 13 pairs of pleiotropic epistatic QTLs that were detected between QTLs located on different chromosomes, such as 1 and 6, 3 and 12, and 5 and 18, across traits, years and populations.

### Candidate gene prediction and gene ontology (GO) enrichment analysis

In order to determine the candidate genes affecting each trait, we investigated six promising genomic regions (*q4–1, q4–2, q11–1, q13, q18*, and *q20*) based on the annotation of soybean reference genome *W82.a2.v1*, which have larger *r*^2^ values and LOD scores that were stably expressed across environments (Table 2). A total of 60, 98, 128, 56, 23, and 401 annotated genes were identified for *q4–1*, *q4–2*, *q11–1*, *q13*, *q18*, and *q20*, respectively. (Table S6). Gene ontology (GO) enrichment analysis showed that the main enriched GO terms were cellular process, protein metabolic process, protein modification process, macromolecule modification, nucleoside binding, lipid binding, ATP binding, phosphorylation, phosphate metabolic process, malate transport, C4-dicarboxylate transport, pigment biosynthetic process, oxidoreductase activity, kinase activity, transferase activity, and methylation (Table S7).

To further explore the promising candidate genes for specific traits, we focused on those genes related to LL, LW, photosynthesis and yield related traits in the six major loci. For example, the *q20* locus, which is located in an region of approximately 4.6-Mb and was previously found to be associated with leaf shape traits using GWAS (Fang et al. 2017), contains several predicted genes encoding proteins that might be involved in regulating leaf size and shape and photosynthetic metabolic processes: narrow leaflet (*Glyma.20G116200*), WUSCHEL related homeobox 13 (*Glyma.20G099400*), phototropic-responsive NPH3 family protein (*Glyma.20G133100*), photosystem I subunit D-2 (*Glyma.20G144700*), translocon at inner membrane of chloroplast (*Glyma.20G129100*), chloroplast biosynthetic enzyme (*Glyma.20G142000*), and chlorophyll A-B binding family protein (*Glyma.20G150600*). previously.

Among these above-mentioned genes, *Glyma.20G116200* has been reported as a key regulator of leaflet shape and number of seeds per pod in soybean [16], and the *Glyma.20G099400* was significantly up-regulated (5.7-fold) in the leaves of the narrow-leaf and high light efficiency genotype Nannong94–156 compared with Bogao based on transcriptome analysis [17]. Another novel locus on chromosome 4, *q4–1*, associated with leaf-related traits, CC and 100-SW was mapped to an approximately 4.0-Mb genomic region. There were 98 annotated genes (Table S6) predicted in this region, incuding one encoding photosystem II reaction center protein D (*Glyma.04G095000*) and one encoding photosystem I subunit G (*Glyma.04G112800*). The major locus, *q4–2*, associated with leaf-related traits and CC was mapped to an approximately 3.5-Mb genomic region on chromosome 4. This region contains 128 annotated genes (Table S7), and two of them encode cellulose synthase 6 (*Glyma.04G173700*), and light-harvesting chlorophyll-protein complex (*Glyma.04G167900*).

## Discussion

The growth and productivity crops depend on photosynthesis, which in turn are largely influenced by both leaf-related traits and CC [4]. However, both leaf and photosynthetic-related traits are typical complex quantitative traits, which are easily influenced by environment and may have epistatic effect. Therefore, the genetic basis of leaf-related traits and CC is still incomplete, especially the genetic relationship between these traits is surprisingly understudied. Most previous studies have focused on discrete analysis of individual traits in a single mapping population, and were limited in their ability to provide a comprehensive analysis for the genetic structure of complex quantitative traits [18, 19]. Another constraint may be that only a part of the genetic structure of traits could be revealed by using the single bi-parental mapping populations, and prevent the excavation of specific favorable alleles [20, 21]. One effective approach is to integrate different metrics (correlation analysis, principal component analysis and genetic analysis) into a low dimensional framework to identify the phenotypic relationship between leaf-related traits and CC [22]. In addition to this method, by analyzing multiple populations with a wider range of genetic variation samples, the power to dissect the genetic structure of quantitative traits could be enhanced.

In this study, we used such an approach to dissect the genetic basis of chlorophyll and leaf-related traits and the relationships between them in soybean. We selected three representative RIL populations, which have high-density molecular marker in genetic maps, to provide a guarantee for the fine mapping of target QTLs and map-based cloning. Phenotypic analysis showed that the six parents and their derived populations exhibited high levels of genetic diversity and significant genetic variation in leaf-related traits, CC, and 100-SW when grown in the field (Fig. 1, Table S1). For example, extensive variation exists for LL (range is from 7.03 to 18.30 mm), LW (2.53 to 11.40 mm), LA (17.69 to 166.76 mm^2^) and CC (7.19 to 53.23) across the three RIL populations (Table S1). The large phenotypic variation of the complex quantitative traits within the RIL populations ensures efficient dissection of the genetic structure of these traits and the determination of major and stable genome regions. In addition, the leaf-related traits were highly correlated with each other, and moderately correlated with CC, which suggests that the functional genes controlling these traits may be closely associated to some extent or pleiotropic. Moreover, the close phenotypic relationship of leaf-related traits and CC with yield related traits was revealed by PCA across the three RIL populations, indicating that these traits are controled by complex and interrelated genetic components (Fig. 3 and Table S3).

In this study, the overlap between QTLs further supports the close genetic relationship between leaf-related traits and CC (Fig. 4, Table 2, and Table S4). We found that even when different traits were analyzed separately, the QTLs of leaf-related traits and CC were frequently co-localized in different RIL populations, suggesting that common genetic components were the basis of observed phenotypic variation. A considerable proportion of leaf-related QTLs (40%, 10 of 25 loci) overlapped with CC QTLs (Table 2 and Table S4), including four major QTL clusters for both traits (Fig. 4). It is noteworthy that the relationship between QTL clusters for leaf-related traits and CC may correspond to control by pleiotropic genes. Overall, the significant phenotype correlation and the identification of co-localized QTLs provide evidence for the close genetic relationship between leaf-related traits and CC. In addition, considering that the chlorophyll may be affected by the plant maturity, we compared the location of these CC QTLs with major genes/QTL for maturity date from other studies. We found that several maturity related QTLs, such as *reproductive period 4-g5*, and *reproductive period 4-g9* were co-localized with CC QTLs in our study (Table S4), suggesting that CC may be related to maturity date. In fact, our previous experimental results also proved this point, so we selected chlorophyll content at R6 in this study, mainly because we found that the chlorophyll in R6 had a greater impact on yield.

As early as the 1960s, the ideal wheat plant was described as having small, erect leaves [23]. In soybean, it has been reported that under dense planting conditions, long and small leaves capture more light energy than round leaves, which is beneficial to the utilization of light energy by the population [24]. But at present, the underlying genetic mechanism of the ideal plant architecture for light energy utilization is not clear. In our study, favorable alleles responsible for most overlapping QTLs came from the male parents, Dongnong50 (DN50), Suinong 14 and Nannong 94–156 (NN94156), which had larger L/W ratios (~ 3.0) than the female parents (~ 1.5). Interestingly, we found that parents with larger L/W ratios tended to have higher CC (Table S1). Moreover, QTL analysis revealed that most of the alleles with positive additive effects on CC and 100-SW were also derived from the male parents (Table S4). These results may provide the genetic basis where the ideal soybean plant architecture requires pointed leaves that are linear and small, which is more conducive to ventilation and light transmission. The selection of genotypes with larger LL or L/W ratio may be a potential approach to improve soybean plant architecture, photosynthetic efficiency, and even yield.

Plant growth and development is a very complex process, which is affected by the genotype, environment and the interaction between them [25]. As an important factor affecting phenotype, QTL × environment interaction may explain one of the reasons why QTLs can not be identified stably in different environments [26, 27]. Previously, many studies have shown complex quantitative traits were controlled by both genetic and environmental factors in soybean [19, 28, 29]. In this study, there were significant differences in phenotypic values across genotypes, years and populations, suggesting that the leaf-related traits and CC are both influenced by the underlying genes, the environment, and different hereditary backgrounds (Table S1). According to our expectation, 10 QTLs were detected only in one mapping population, and five QTLs were detected only in one environment, suggesting that the genetic basis of leaf-related traits and CC are partly affected by the environment (Fig. 4, Table 2 and Table S4). This result is largely similar with the report that there is a interaction between leaf traits and the environment [18]. Furthermore, the differences in the distribution of QTLs across the populations, show that it is the key to dissect the genetic structure to determine the background effect by analyzing multiple populations.

Another important contributor to the genetic structure of quantitative traits is epistasis, which has been reported to play an important role controlling LA in maize [30]. In the present study, 74 additive×additive epistatic interactions were detected for the six traits. The phenotypic contribution rate for these epistatic QTLs was 9.83% on average and it ranged from 2.22 to 19.25% (Fig. 4, Table S5), showing that epistatic may play an considerable role in the inheritance of soybean leaf-related traits and CC. Compared with other studies, we detected more epistatic QTLs, which may be because genetic analysis was performed using multiple populations grown in multiple environments and was based on high-density genetic maps.

In this study, QTLs were identified on almost all the chromosomes, but those on chromosomes 4, 11, 13, 18, and 20 had the largest and most consistent effects on leaf-related traits and CC (Table 2 and Fig. 4). Moreover, the major QTLs for leaf-related traits and CC were co-localized (*q4–1*, *q4–1, q13* and *q20*) or specific (*q11* and *q18*) (Table 2 and Fig. 4). For example, the major QTL, *q20*, was co-localized to previously identified loci related to LL, LW, LA, leaf shape and seed set [14], plant height [31], and branch number [32], water use efficiency [33], and shoot phosphorus content [34], indicating the presence of important genes in this region may be involved in regulating soybean plant architecture and even yield. More importantly, we also found that *q20* was co-localized with *Ln*, which is a key regulator of leaflet shape in soybean [19]. To further analyze the relationship between *Ln* gene and the leaf related traits in our study, we conducted a partial single marker analysis at the *Ln* locus by using investigating the association of all the molecular markers (45 SNPs) distributed in the range of within 1 Mb upstream and downstream of *Ln* locus with the leaf related traits, including leaf width (LW), leaf length (LL), and leaf area (LA) and chlorophyll content (CC). The results showed that the markers adjacent with *Ln* gene were significantly correlated (*p* < 10^− 5^) with leaf related traits (especially for LW), which strongly suggest that *Ln* may be a candidate for the major QTL, *q20*. In addition, we also found a single nucleotide substitution (G/C) in the coding region of the *Gm-JAG1* gene which led to a change of single amino acid based on the sequencing data. This allelic variation was corresponding to the leaf type of the parent, including G-type for W82, Enrei and Bogao, C-type for DN50, SN14, and NN94–156.

Interestingly, we found that *q20* controlled both leaf-related traits and CC in the S × E population across years; ours is the first study to find that there are QTLs related to photosynthesis in this locus. In addition, a putative gene encoding WUSCHEL related homeobox 13 (*Glyma.20G099400*) was considered a possible candidate in this region because it is generally believed to be critical for leaf shape and leaf development in plants, such as in *Arabidopsis* [35], rice [36, 37], *Medicago* [38], and azalea [39]. Futhermore, our previous expression analysis indicated that the expression of *Glyma.20G099400* in a narrow-leaf and high light efficiency parent genotype (N) was significantly higher than that in Bogao based on transcriptome analysis (Zhang et al. 2017). The expression level of *Glyma.20G099400* in Nannong94–156 was significantly increased, indicating that it may be involved in leaf development and photosynthesis. Therefore, *Glyma.20G099400* was considered to be involved in the regulation of the two traits, which is worth further experimental verification.

A novel QTL, *q4–2*, was mapped detected on chromosome 4 for both leaf-related traits and CC, suggesting that this QTL is pleitropic, further demonstrating the physiological association between leaf-related traits and CC. A promising putative gene (*Glyma.04G173700*) underlying *q4–2*, which encodes cellulose synthase, has been previously identified to play an important role in leaf development in rice [40, 41], maize [42] and broccoli [43]. In addition, we also found several predicted genes in this genetic region and other major QTL regions, including those encoding a phototropic-responsive NPH3 family protein, photosystem I subunit D-2, a translocon at inner membrane of chloroplast, a chloroplast biosynthetic enzyme, and a chlorophyll A-B binding family protein, and it may be involved in the leaf development and photosynthetic metabolism processes (Table S6).

## Conclusion

In conclusion, despite the high complexity of leaf-related traits and CC, our results shows that there is a close genetic relationship between the major QTLs controlling both trait. These results provide a new perspective for better understanding of the genetic basis for leaf-related traits and CC, which could be used to produce soybean genotypes with the ideal plant architecture and efficient photosynthesis. These major loci, *q4–1*, *q4–2*, *q11, q13, q18* and *q20* and its candidate genes or the SNP markers closely linked to these QTLs could be further used in molecular breeding (genetic engineering breeding, molecular marker assisted selection breeding). Overall, our finding simultaneously using leaf-related trait and CC data to study intensively the genetic relationship between plant architecture and photosynthesis in multiple environments and soybean populations.

## Methods

### Plant materials

Three RIL populations with distinct leaf shapes were used to identify QTLs controlling leaf-related traits and CC (Table 1). The first segregating population consisted of 152 F_12–13_ lines derived from a cross (N × B) combination between Nannong94–156 (male parent) [44], which possesses relatively narrow, long leaves and short internodes, and Bogao (female parent) (N × B) with round, short leaves and longer internodes. Both parental lines exhibited significant variation in photosynthetic-related traits and plant height in our previous studies [13]. The second RIL population consisted of 127 F_11–12_-derived lines and was derived from a cross (D × W) between Dongnong50 (female parent) and Williams82 (male parent) using the single-seed descent method [45]. Dongnong50 is a small-leaf variety introduced from Canada, and has a smaller leaf size and taller plant height than Williams82. The third RIL population consisted of 154 F_12–13_-derived lines from a cross (S × E) between Suinong14 × Enrei [45]. Suinong14 is an early-maturing spring soybean cultivar grown in Northern China with a narrow leaf and a high chlorophyll content, whereas Enrei is one of the most common Japanese cultivars with a wide leaf. The genomes of all six parents have been sequenced, which laid the foundation for candidate gene mining within the QTL region.

### Field experiments

All three populations along with their parents were evaluated in field trials across two environments in China. The N × B population was grown at Maozhuang Experimental Station in Zhengzhou in the 2018 cropping season and at Yuanyang in Henan province in the 2019 cropping season; the D × W and S × E populations were grown at Yuanyang in Henan in the 2018 and 2019 cropping seasons. A randomized block design with three replicates was employed in the field trials, and each line was planted in three rows, has a row length of 200 cm and a row spacing of 60 cm. Agronomic management was conducted according to local customs in each location.

### Phenotype measurement

Five plants were selected from the middle row as samples for measuring LL, LW, LA, L/W, CC and 100-SW in each plot. According to our previous research results on the QTLs controlling chlorophyll content during different developmental stages (Cui and Yu 2007), we found that CC in the full seed reproductive growth stage (R6) has a greater impact on yield, so we meansured leaf-related traits and CC at R6 in this study. The upper third leaf from five plants per line for each of three replications was used for phenotyping at the R6 stage. Briefly, CC was measured by chlorophyll meter from 9 am to 11 am (CCM-200, OptiSciences, Inc., USA). Then the leaf samples were immediately collected and stored at 4 °C until leaf-related traits (LL, LW, LA and L/W) were measured using an STD 4800 scanner (Epson, Japan). Image analysis software based on MATLAB 2013a, LEAFAREAS2.0 (http://pan.baidu. com/s/1c07vkGS), was used to obtain phenotypic values for LL, LW, LA and L/W. All lengths are reported in centimeters. 100-SW was determined by counting and weighing 100 seeds form each sample, and is reported in grams.

### High-density genetic maps

In this study, a high-density linkage map of the N × B population was reported previously by Zhang et al. (2016). This linkage map contained 6159 SNP markers, and the average distance between adjacent markers is 0.49 cM [12]. Linkage maps of the other two populations (D × W and S × E) were both provided by Guangzhou University; these genetic maps were constructed using the genotyping by sequencing method, and comprise 2015 and 5660 bin markers, respectively. Briefly, for these two linkage maps, we use the IciMapping V4.0 software [15] to place SNP markers without recombination into a bin; next, ues JoinMap 4.0 [46] to divide the frame markers into each groups with a LOD threshold ≥7; last, use MapDisto 1.7 [47] to order the frame markers and then calculate the genetic distance; finally, the linkage groups were assigned and oriented on chromosomes based on the SoyBase (https://www.soybase.org/).

### Statistical analysis

Analysis of variance (ANOVA) of phenotypic data was performed by using the general linear model (GLM) procedure in SPSS Statistics 17.0 (SPSS, Inc., Chicago, IL, USA). Genotype (G) was treated as fixed, combinations of year-location were considered environments (E), and the genotype-by-environment (G × E) interaction was treated as random. Pearson correlation coefficients for these traits were calculated based on mean values by using SPSS 17.0 and further visualized using the R package. Graphpad prism 7.0 were used to creat the frequency distribution graphs. Broad-sense heritabilities (*h*^2^) were analyzed using *h*^2^ = Vg/ (Vg + Ve). Vg and Ve indicate genetic variation (extracted from the ANOVA results) and environmental variation, respectively.

### QTL identification and comparison

The identification of additive and epistatic QTLs for the leaf related-traits and CC were performed using single environment phenotypic values across different environments by the QTL IciMapping program v4.0 [48]. The parameter settings of composite interval mapping (ICIM) and ICIM-EPI method was described as previous reports [16]. Briefly, the ICIM method was used to map the additive QTL, the *P* values for entering variables (PIN) and removing variables (POUT) were set at 0.01 and 0.02, and the scanning step was 2 cM. Positions were estimated from peaks having the LOD score over the predefined threshold of 2.5. The ICIM-EPI method was used to detect epistatic QTL, the PIN and POUT were set at 0.0001 and 0.0002, respectively, and the scanning step was 5 cM. The phenotypic variance explained (PVE) by each additive QTL or epistatic QTL and the corresponding additive effects were also estimated.

In order to better reveal the genetic mechanism of leaf related traits and chlorophyll, the physical positions QTL for the same traits were compared in different populations based on the physical location of the *G. max* reference genome (Wm82.a2.v1). The categorization of QTLs mainly follows the following two rules: 1) the QTL (the distance between the LOD contour peaks is less than 5 cM) for one trait detected across environments and populations were considered to be the same; 2) QTL could be identified more than five times across years, traits or populations were considered to be major and stable.

### Candidate gene discovery and gene ontology (GO) enrichment analysis

In the target QTL regions, firstly, the candidate genes were predicted based on the annotation of the soybean reference genome in Phytozome v.12 (https://phytozome.jgi.doe.gov). Then the annotation functional of candidate genes were compared manually in NCBI by blastp function. Finally, we performed Gene Ontology (GO) enrichment analysis online (http://bioinfo.cau.educn/agriGO/ analysis.php) of these predicted genes.

## Supplementary information

**Additional file 1: Figure S1.** Phenotypic distribution of the six traits across environments. BG, SE, and WD denote the mapping populations in the corresponding environments.

**Additional file 2: Table S1.** Trait variation and heritability in the parental lines and three RIL mapping populations**. Table S2.** Pearson’s correlation coefficients (r) between all investigated traits of RILs across years and three mapping populations**. Table S3.** Principal component analysis (PCA) on the three RIL mapping populations. **Table S4.** Summary of detected QTL for all investigated traits in the three RIL populations acoss years. **Table S5.** Epistasis QTL for the investigated traits in the three RIL populations acoss years. **Table S6.** Predicted candidate genes associated with leaf related traits and chlorophyll content in six major QTL regions. **Table S7.** Gene Ontology (GO) enrichment analysis of six major QTLregoins associated with leaf related traits and chlorophyll content.

## Data Availability

The data supporting the conclusions of this study are within the paper and. its additional files.

## Abbreviations

W × D: Williams82 × Dongnong50
S × E: Suinong14 × Enrei
N × B: Nannong94–156 × Bogao
LL: leaf length
LW: leaf width
LA: leaf area
L/W: the ratio of leaf length and width
CC: chlorophyll content
100-SW: 100 seed weight
RILs: recombinant inbred lines
G: genotype
E: environments
G × E: genotype-by-environment
ANOVA: analysis of variance
GLM: general linear model
*h*^2^: broad-sense heritabilities
QTL: quantitative trait locus
LOD: logarithum of the odds
ICIM: inclusive composite interval mapping
ICIM-EPI: inclusive composite interval mapping-epistatic
SNP: single nucleotide polymorphisms
PCA: principal component analysis
GWAS: genome-wide association studies
GO: gene ontology

## References

[1] Sarlikioti V, de Visser PHB, Buck-Sorlin GH, Marcelis LFM (2011). How plant architecture affects light absorption and photosynthesis in tomato: towards an ideotype for plant architecture using a functional-structural plant model. Ann Bot.

[2] Thompson JA, Nelson RL, Schweitzer LE (1995). Relationships among specific leaf weight, photosynthetic rate, and seed yield in soybean. Crop Sci.

[3] Ma BL, Morrison MJ, Voldeng HD (1995). Leaf greenness and photosynthetic rates in soybean. Crop Sci.

[4] Sakowska K, Alberti G, Genesio L, Peressotti A, Vedove GD, Gianelle D, Colombo R, Rodeghiero M, Panigada C, Juszczak R. Leaf and canopy photosynthesis of a chlorophyll deficient soybean mutant. Plant Cell Environ. 2018;41:1427–37. 10.1111/pce.13180 29498070

[5] Curran PJ, Windham WR, Gholz HL (1995). Exploring the relationship between reflectance red edge and chlorophyll concentration in slash pine leaves. Tree Physiol.

[6] Todeschini MH, Milioli AS, Rosa AC, Dallacorte LV, Panho MC, Marchese JA, Benin G (2019). Soybean genetic progress in South Brazil: physiological, phenological and agronomic traits. Euphytica.

[7] Takai T, Kondo M, Yano M, Yamamoto T (2010). A quantitative trait locus for chlorophyll content and its association with leaf photosynthesis in Rice. Rice.

[8] Hou MJ, Tian F, Zhang T, Huang MS (2019). Evaluation of canopy temperature depression, transpiration, and canopy greenness in relation to yield of soybean at reproductive stage based on remote sensing imagery. Agric Water Manag.

[9] Liu X, Liu LL, Xiao YH, Liu SJ, Tian YL, Chen LM, Wang ZQ, Jiang L, Zhao ZG, Wan JM (2015). Genetic dissection of leaf-related traits using 156 chromosomal segment substitution lines. J Plant Biol.

[10] Quarrie S, Pekic QSR, Rancic D, Kaminska A, Barnes JD, Leverington M, Ceoloni C, Dodig D (2006). Dissecting a wheat QTL for yield present in a range of environments: from the QTL to candidate genes. J Exp Bot.

[11] Jia H, Wan H, Yang S, Zhang Z, Kong Z, Xue S, Zhang L, Ma Z (2013). Genetic dissection of yield-related traits in a recombinant inbred line population created using a key breeding parent in China’s wheat breeding. Theoretical Applied Genetics.

[12] Zhang D, Li H, Wang J, Zhang H, Hu Z, Chu S, Lv H, Yu D. High-density genetic mapping identifies new major loci for tolerance to low-phosphorus stress in soybean. Front Plant Sci. 2016;7:372. 10.3389/fpls.2016.00372 PMC4811872 27065041

[13] Li HY, Yang YM, Zhang HY, Chu SS, Zhang XG, Yin DM, Yu DY, Zhang D. A genetic relationship between phosphorus efficiency and photosynthetic traits in soybean as revealed by QTL analysis using a high-density genetic map. Front Plant Sci. 2016;7:924. 10.3389/fpls.2016.00924 PMC4923142 27446154

[14] Fang C, Ma YM, Wu SW, Liu Z, Wang Z, Yang R, Hu GH, Zhou ZK, Yu H, Zhang M, Pan Y, Zhou GA, Ren HX, Du WG, Yan HR, Wang YP, Han DZ, Shen YT, Liu SL, Liu TF, Zhang JX, Qin H, Yuan J, Yuan XH, Kong FJ, Liu BH, Li JY, Zhang ZW, Wang GD, Zhu BG, Tian ZX. Genome-wide association studies dissect the genetic networks underlying agronomical traits in soybean. Genome Biology. 2017;18:161. 10.1186/s13059-017-1289-9 PMC5571659 28838319

[15] Jun TH, Freewalt K, Michel AP, Mian R (2014). Identification of novel QTL for leaf traits in soybean. Plant Breed.

[16] Jeong N, Suh SJ, Kim M-H, Lee S, Moon J-K, Kim HS, Jeong S-C (2012). Ln is a key regulator of leaflet shape and number of seeds per pod in soybean. Plant Cell.

[17] Zhang D, Zhang H, Chu S, Li H, Chi Y, Triebwasser-Freese D, Lv H, Yu D (2017). Integrating QTL mapping and transcriptomics identifies candidate genes underlying QTLs associated with soybean tolerance to low-phosphorus stress. Plant Mol Biol.

[18] Cui T, Kunhui H, Liguo C, Xinghua Z, Jiquan X, Jianchao L (2017). QTL mapping for leaf area in maize (*Zea mays* L.) under multi-environments. J Integr Agric.

[19] Wang L, Cheng Y, Ma Q, Mu Y, Huang Z, Xia Q, Zhang G, Nian H. QTL fine-mapping of soybean (*Glycine max* L.) leaf type associated traits in two RILs populations. Bmc Genomics. 2019;20:260. 10.1186/s12864-019-5610-8 PMC6444683 30940069

[20] Liller CB, Walla A, Boer MP, Hedley P, Macaulay M, Effgen S, von Korff M, van Esse GW, Koornneef M (2017). Fine mapping of a major QTL for awn length in barley using a multiparent mapping population. Theoretical Applied Genetics.

[21] Cavanagh C, Morell M, Mackay I, Powell W (2008). From mutations to MAGIC: resources for gene discovery, validation and delivery in crop plants. Curr Opin Plant Biol.

[22] Gegas VC, Nazari A, Griffiths S, Simmonds J, Fish L, Orford S, Sayers L, Doonan JH, Snape JW (2010). A genetic framework for grain size and shape variation in wheat. Plant Cell.

[23] Donald CM (1968). The breeding of crop ideotypes. Euphytica.

[24] Rongwen J, Akkaya MS, Bhagwat AA, Lavi U, Cregan PB (1995). The use of microsatellite DNA markers for soybean genotype identification. Theoretical Applied Genetics.

[25] El-Soda M, Malosetti M, Zwaan BJ, Koornneef M, Aarts MG (2014). Genotypexenvironment interaction QTL mapping in plants: lessons from *Arabidopsis*. Trends Plant Sci.

[26] Boer MP, Deanne W, Lizhi F, Podlich DW, Lang L, Mark C, Eeuwijk FA (2007). Van, a mixed-model quantitative trait loci (QTL) analysis for multiple-environment trial data using environmental covariables for QTL-by-environment interactions, with an example in maize. Genetics.

[27] Veldboom LR, Lee M (1996). Genetic apping of qunatitative trait loci in maize in stress and nonstress environments: II. Plant height and flowering. Crop Sci.

[28] Zhang D, Zhang HY, Hu ZB, Chu SS, Yu KY, Lv LL, Yang YM, Zhang XQ, Chen X, Kan GZ, Tang Y, An YQCRL, Yu DY. Artificial selection on GmOLEO1 contributes to the increase in seed oil during soybean domestication. Plos Gen. 2019;15:e1008267. 10.1371/journal.pgen.1008267 PMC6645561 31291251

[29] Zhang D, Song HN, Cheng H, Hao DR, Wang H, Kan GZ, Jin HX, Yu DY. The acid phosphatase-encoding gene GmACP1 contributes to soybean tolerance to low-phosphorus stress. Plos Gen. 2014;10:e1004061. 10.1371/journal.pgen.1004061 PMC3879153 24391523

[30] Iqbal M, Khan K, Rahman H, Sher H (2010). Detection of epistasis for plant height and leaf area per plant in maize (*Zea mays* L.) from generation means analysis. Maydica.

[31] Zhang J, Song Q, Cregan PB, Nelson RL, Wang X, Wu J, Jiang GL (2015). Genome-wide association study for flowering time, maturity dates and plant height in early maturing soybean (*Glycine max*) germplasm. BMC Genomics.

[32] Zhang H, Hao D, Sitoe HM, Yin Z, Hu Z, Zhang G, Yu DJPB (2015). Genetic dissection of the relationship between plant architecture and yield component traits in soybean (Glycine max) by association analysis across multiple environments. Plant Breed.

[33] Kumar M, Lal SK (2015). Molecular analysis of soybean varying in water use efficiency using SSRs markers. J Environ Biol.

[34] Dhanapal AP, Ray JD, Smith JR, Purcell LC, Fritschi FB. Identification of novel genomic loci associated with soybean shoot tissue macro- and micronutrient concentrations. Plant Genome. 2018;11(2):170066. 10.3835/plantgenome2017.07.0066 PMC12962512 30025027

[35] Wang J, Tian CH, Zhang C, Shi BH, Cao XW, Zhang TQ, Zhao Z, Wang JW, Jiao YL (2017). Cytokinin signaling activates WUSCHEL expression during axillary meristem initiation. Plant Cell.

[36] Wu C, Fu YP, Hu GC, Si HM, Cheng SH, Liu WZ (2010). Isolation and characterization of a rice mutant with narrow and rolled leaves. Planta.

[37] Cho S-H, Yoo S-C, Zhang H, Pandeya D, Koh H-J, Hwang J-Y, Kim G-T, Paek N-C (2013). The rice narrow leaf2 and narrow leaf3 loci encode WUSCHEL-related homeobox 3A (OsWOX3A) and function in leaf, spikelet, tiller and lateral root development. New Phytol.

[38] Meng Y, Liu H, Wang H, Liu Y, Zhu B, Wang Z, Hou Y, Zhang P, Wen J, Yang H, Mysore KS, Chen J, Tadege M, Niu L, Lin H (2019). HEADLESS, a WUSCHEL homolog, uncovers novel aspects of shoot meristem regulation and leaf blade development in Medicago truncatula. J Exp Bot.

[39] Tasaki K, Nakatsuka A, Cheon KS, Kobayashi N. Genetic demonstration of the involvement of WUSCHEL-related homeobox (WOX) genes in narrow-petal and narrow-leaf mutations in traditional Japanese azalea cultivars. Euphytica. 2019;215(1):5.

[40] Ding ZQ, Lin ZF, Li Q, Wu M, Xiang CY, Wang JF (2015). DNL1, encodes cellulose synthase-like D4, is a major QTL for plant height and leaf width in rice (*Oryza sativa* L.). Biochem Biophysical Res Communications.

[41] Yoshikawa T, Eiguchi M, Hibara K-I, Ito J-I, Nagato Y (2013). Rice SLENDER LEAF 1 gene encodes cellulose synthase-like D4 and is specifically expressed in M-phase cells to regulate cell proliferation. J Exp Bot.

[42] Hunter CT, Kirienko DH, Sylvester AW, Peter GF, McCarty DR, Koch KE (2012). Cellulose synthase-like D1 is integral to Normal cell division, expansion, and leaf development in maize. Plant Physiol.

[43] Li S, Zhang L, Wang Y, Xu F, Liu M, Lin P, Ren S, MaR, Guo YD. Knockdown of a cellulose synthase gene BoiCesA affects the leaf anatomy, cellulose content and salt tolerance in broccoli. Sci Rep. 2017;7:41397. 10.1038/srep41397 PMC5294630 28169290

[44] Zhang D, Cheng H, Geng L, Kan G, Cui S, Meng Q, Gai J, Yu D (2009). Detection of quantitative trait loci for phosphorus deficiency tolerance at soybean seedling stage. Euphytica.

[45] Kong L, Lu S, WangY, Fang C, Wang F, Nan H, Su T, Li S, Zhang F, Li X, Zhao X, Yuan X, Liu B, Kong F. Quantitative trait locus mapping of flowering time and maturity in soybean using next-generation sequencing-based analysis. Front Plant Sci. 2018;9:995. 10.3389/fpls.2018.00995 PMC6050445 30050550

[46] Stam P (1993). Construction of integrated genetic linkage maps by means of a new computer package: join map. Plant J.

[47] Lorieux M (2012). MapDisto: fast and efficient computation of genetic linkage maps. Mol Breed.

[48] Meng L, Li H, Zhang L, Wang J (2015). QTL IciMapping: integrated software for genetic linkage map construction and quantitative trait locus mapping in biparental populations. Crop J.

