# Supplementary figures and images for: High-density QTL mapping of leaf-related traits and chlorophyll content in three soybean RIL populations
Source: BMC Plant Biol. 2020 Oct 13;20:470. doi: 10.1186/s12870-020-02684-x (PMC7556954; doi:10.1186/s12870-020-02684-x)

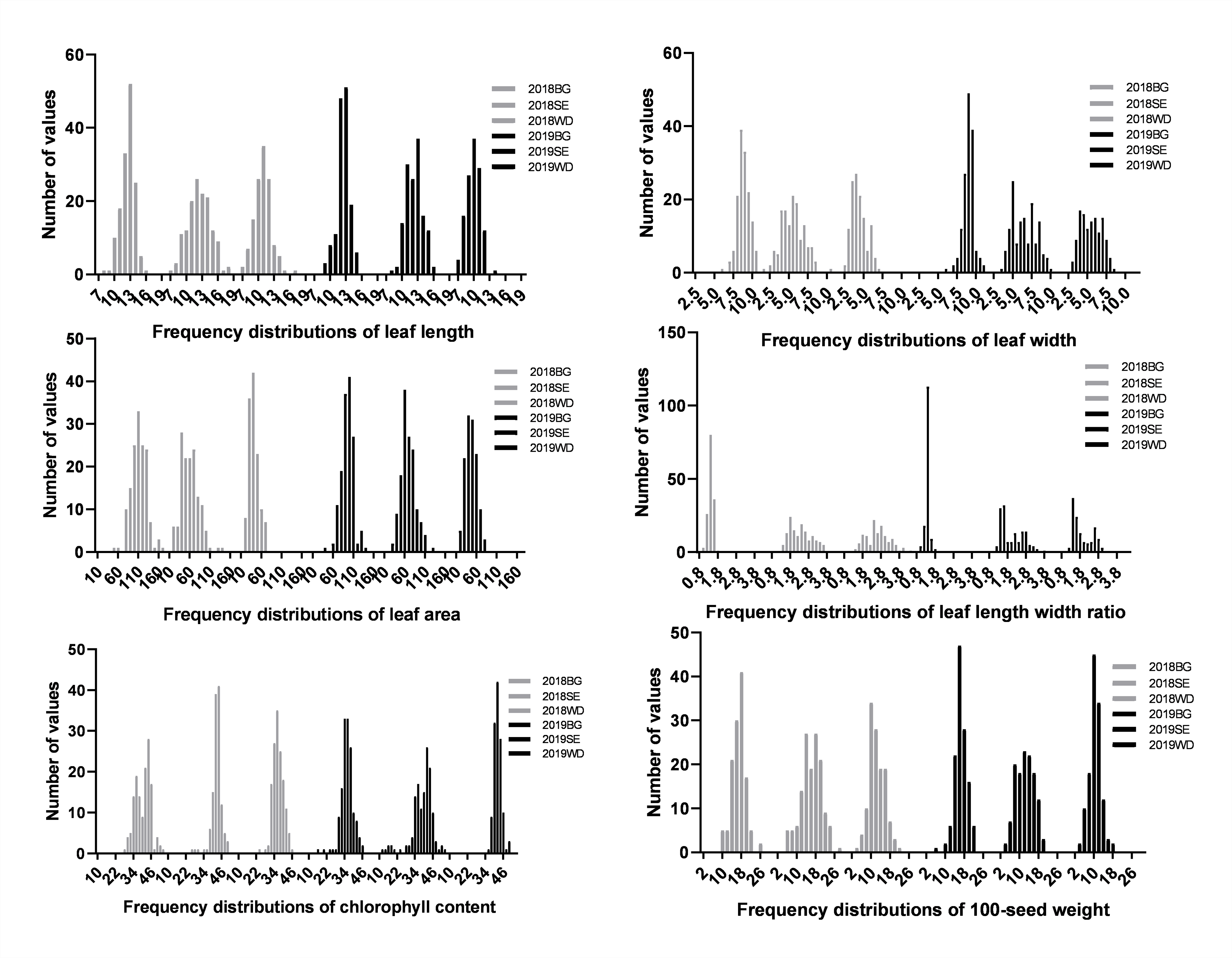

Supplement: Supplementary file 1 — Additional file 1: Figure S1. Phenotypic distribution of the six traits across environments. BG, SE, and WD denote the mapping populations in the corresponding environments. [file 12870_2020_2684_MOESM1_ESM.tif]
